# A health communication campaign for prevention of osteoporosis in rural elderly women

**DOI:** 10.1186/s12905-023-02282-7

**Published:** 2023-03-23

**Authors:** Solmaz Dastmanesh, Masoud Karimi, Leila Ghahremani, Mozhgan Seif, Elahe Zare

**Affiliations:** 1 Research Center for Health Sciences, Institute of Health, Department of Health Promotion, School of Health, Shiraz University of Medical Sciences, Shiraz, Iran; 2 Non-Communicable Diseases Research Center, Department of Epidemiology, School of Health, Shiraz University of Medical Sciences, Shiraz, Iran, Shiraz University of Medical Sciences, Shiraz, Iran; 3 MSc, Research Center for Health Sciences, Institute of Health, Department of Health Promotion, School of Health, Shiraz University of Medical Sciences, Shiraz, Iran

**Keywords:** Knowledge, Attitude, Practice, Elderly women, Osteoporosis

## Abstract

**Background:**

Osteoporosis, as the most common metabolic disease and the leading cause of death among older people, affects more than 200 million women throughout the world. This study aimed to evaluate the effect of a health communication campaign on knowledge, attitude, and practice of older women towards prevention and control of osteoporosis.

**Methods:**

In this multi-stage mixed methods study, 30 days’ health communication campaign for prevention of osteoporosis was conducted on 60- 75-year-old women, in rural areas of Fasa, Iran. Subjects were divided into two groups, control (n = 103) and intervention (n = 98). Data were collected using a researcher-made questionnaire and analyzed by SPSS 25.0. The significance level was set at < 0.05.

**Results:**

Inter-group group analysis revealed that the mean scores of knowledge, attitude, and practice were not significantly different between the two groups before the intervention, but after the intervention, unlike the behavior (P = 0.569), mean scores of knowledge (p < 0.001) and attitude (p < 0.001) of the intervention group were significantly more than the control group. Intra-group comparisons showed that, unlike the control group, the mean scores of knowledge (p < 0.001), attitudes (p < 0.001), and behavior (P < 0.001) increased significantly in the intervention group.

**Conclusions:**

Health communication campaign is an effective way to change the knowledge and attitude and to a lesser extent the practice of the eldery toward prevention and control of osteoporosis.

## Background

Nowadays, osteoporosis, is an important global public health problem. It is usually asymptomatic and often presents as a clinically evident fracture, so it may impose an increasing physical and economic burden on patients and the society [1]. Osteoporosis is a skeletal system disease characterized by low bone mass and structural deterioration of the bone tissue, resulting in increased bone fragility and risk of fractures [2]. Osteoporosis can be classified into two categories, primary and secondary. Primary osteoporosis can occur in both sexes at any age with aging, while it considerably increases in postmenopausal women and gradually increases among men. Secondary osteoporosis may occur due to certain medications or diseases, such as hyperthyroidism and celiac disease [3].

While Salari et al. (2021) in their meta-analysis study estimated that the prevalence of osteoporosis in women of the world was 23.1% [4], in the last Iranian Multi-center Osteoporosis Study (IMOS), the prevalence of osteoporosis in Iranian postmenopausal women was 37%; about 24.6%, 28.8%, and 5.6% of them suffered from osteoporosis in the femoral neck, spine, and total hip, respectively [5]. Eghbali et al. (2022) in a meta-analysis, which included 26 recent studies in Iran, reported that the prevalence of osteoporosis and osteopenia in Iranian postmenopausal women was 33.70%, and 47.60%, respectively [6]. Another meta-analysis including 30 articles published from 2005 to 2019 reported that the prevalence of osteoporosis in Iranian women over 60 years old was 34% [7].

Women, who make up half of the world’s population are key and productive members of society, and their health affects the health of the subsequent generation. Therefore, preservation of their health is essential. On the other hand, osteoporosis is easily preventable through health education interventions [8].

The risk factors of osteoporosis could be classified as non-modifiable (female sex, advancing age, heredity, and race), and modifiable (poor diet such as reduced calcium intake, insufficient physical activity, smoking, caffeine and alcohol drinking) [9]. However, some studies revealed that the awareness, attitude, and practice of people toward these risk factors were not at desirable levels [10, 11]. The results of IMOS in Iran showed that 81.3% of the females had a poor knowledge of different aspects of osteoporosis and its complications [12]. Thus, it seems necessary to increase health literacy of populations at risk by public health measures.

One of the common health education interventions which made important contributions to the advancement of public health in health systems is health communication campaign [13]. Most of these programs occur with a focus on health issues worldwide. Health communication campaigns (HCC) are “purposive attempts to inform or influence behaviors in large audience within a specified time period, using an organized set of communication activities and featuring specific messages in multiple channels to motivate behavior change in the individual and society” [14].

Because previous studies have shown that osteoporosis is more prevalent in women living in rural areas than those in urban areas [15] and since few studies conducted in Iran have examined the impact of HCCs in urban areas [15, 16]; the present study aimed to evaluate the effects of an educational intervention which was designed and conducted based on HCC principles, on knowledge, attitude, and practice of elderly women towards osteoporosis in rural areas in Fasa, Iran.

## Methods

### Design

In this multi-stage mixed methods study, which lasted six months (June to December 2019) from the time of receiving the code of ethics to completion of data analysis, the intervention was conducted based on a guide entitled “Overview of Health Communication Campaigns” which was developed by The Health Communication Unit (THCU) at the Centre for Health Promotion University of Toronto (2007) [17]. These stages were as follow:

1. Review background information: Investigating the prevalence and the severity of osteoporosis and associated factors based on literature review (mentioned in the introduction section of this article).
2. Set communication objectives: Three specific goals of this project included examining the impact of the communication campaigns on (1) knowledge, (2) attitude, and (3) practice of elderly women.
3. Analyze and segment target audiences: This was done to determine socioeconomic status, lifestyle, and cultural and religious beliefs affecting health of the elderly. For this purpose, three focus groups with totally 7 local health staffs, eight old peoples and, 10 health volunteers were conducted. In this stage, the audience were categorized into primary audience (elderly women) and secondary audience (health volunteers, health staff, and family members of the elderly women).
4. Channel analysis and selection of appropriate communication channel: The aim of this stage was to identify the preferred communication channels of the elderly and determine verbal and non-verbal communication barriers which may cause disruption in communication process at individual and group levels. Since several interpersonal and media channels should be used in HCCs, through consultation with the health education experts and health staff of the studied villages as well as reviewing the literature[18], three categories of communication channels were determined for message transmission to the elderly women, including Mass communication channels (pamphlets, billboards, posters, booklets), Interpersonal channels (Health volunteers, health staffs, physicians), and group channels (walking programs, lectures in mosques, exhibitions).
5. Identify message concepts and pretest: According to the analysis of the audience and channel, proper messages were designed for each target group and channel. The main message and slogan of the campaign was “love our bones”, and “know how to prevent osteoporosis”. The contents of the messages were checked in terms of readability, comprehensibility and in accordance with the cultural values of the community in consultation with local health workers and 10 local trusted health volunteers.
6. Create messages and materials: based on the results of step 5, 1000 pamphlets, 1000 leaflets, 500 booklets, and 15 billboards were produced, mainly for secondary audience, all of which were in the Persian language. A flip chart was designed for use in the educational sessions for the primary audience. It contained a picture or illustration on each paper sheet, so it communicated a single, distinct message in each page.
7. Develop promotion plan: Planning for executive and educational activities was compiled for a 30 days’ period; the time schedule was developed, and one person in charge was appointed for each program. Activities included setting up a health exhibition for three days, in which health services such as face-to-face education and blood pressure, blood glucose level, and body mass index monitoring were provided for the elderly women, holding four 45-minute education sessions for the elderly (especially illiterate ones), in rural health homes, healthcare centers and local mosques, distribution of pamphlets and leaflets, installation of billboards in public places, implementing an education session for health volunteers and holding a one day walking plan for the public population of the villages.
8. Implement communication strategies: Communication campaigns were launched for 30 days, based on the programs which were designed step 7, in the intervention group village. During this time, the control group received routine health education and health care services.
9. Assess effects: The tools and methods of assessments will be explained in detail in the following section, and the related findings are presented in the results section of this paper.

### Sample

**Fig. 1:**
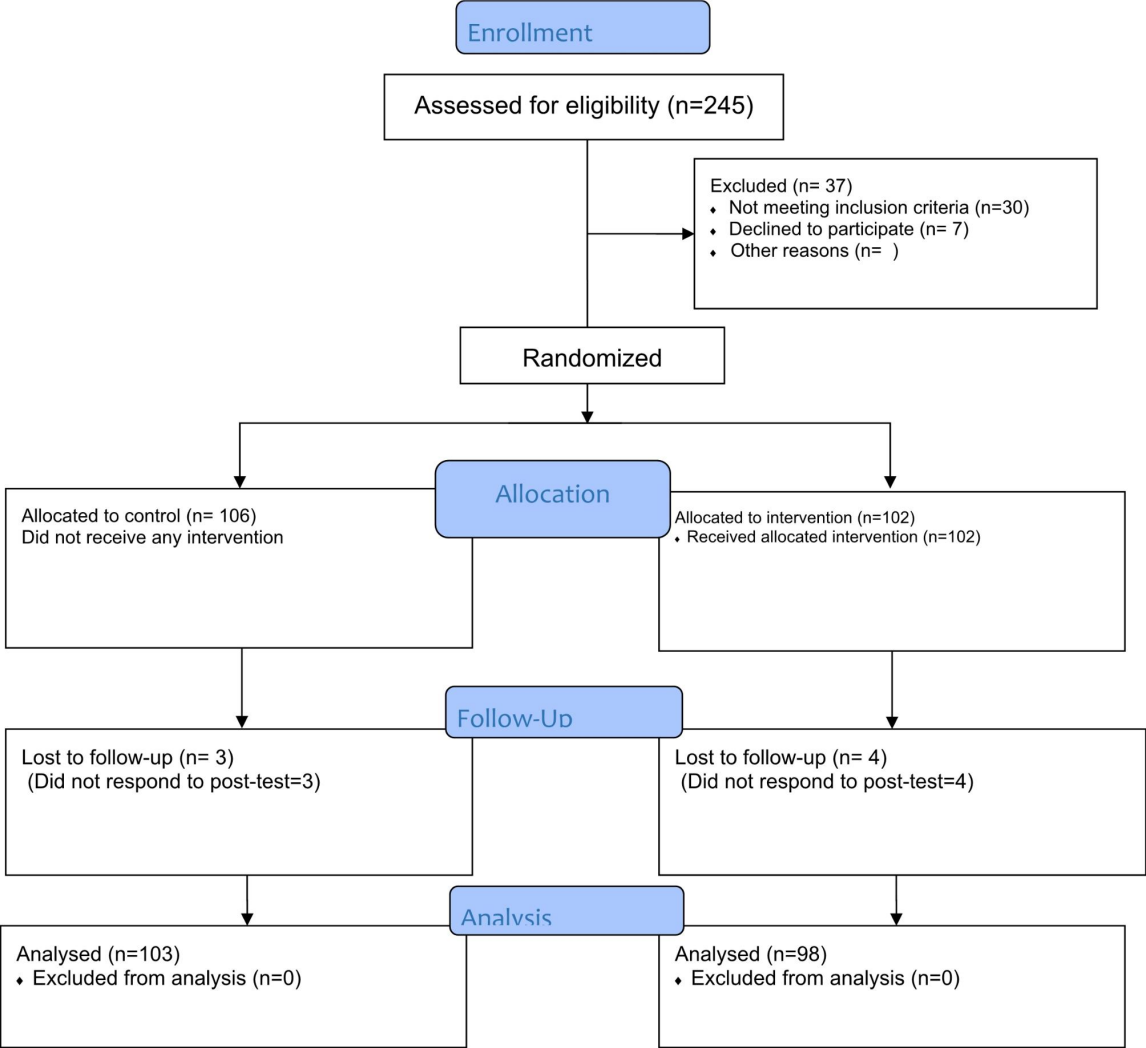
CONSORT flow diagram of participants through the study

Based on a similar study [16] and considering 95% confidence level (α = 0.05), 90% power (1-β = 0.9), and 20% attrition rate, using the NCSS PASS 15 software, the sample size was computed at least 90 participants in each group. For the recruitment of the study population, at first from 55 total villages of Fasa city, seven villages which had at least 100 over 60-year-old female population were detected. After that, in order to ensure that the control group is not exposed to the campaign messages, we divided these villages into two groups based on their geographical location, so that each group was located in one of the geographic poles of the Fasa city with at least 60-kilometer distances. Then, from the villages of each pole, one village was randomly selected. All women aged 60–75 years within the selected villages were recruited for the study. In the next step, the pretest questionnaires were completed; then, the selected villages were allocated into two interventions (102 subjects) and control groups (106 subjects) using simple randomization. Three subjects in the control group and four in the intervention group were excluded from the study due to lost to follow-up (Fig. 1).

The inclusion criteria for the participants in the study were 60-75-year-old women, signing informed consent form to participate in the study, use of the Persian language, lack of severe and debilitating osteoporosis-related complications or other major limiting diseases, such as severe osteoarthritis and heart failure, ability to speak and communicate, and lack of severe cognitive or psychological problems. The participants were excluded from the study if they died, migrated, or traveled during the campaign.

### Measures

Data were collected by a researcher-made questionnaire which consisted of two parts. In the first part, questions about the participants’ demographic information (age, education level), underlying chronic diseases (hypertension, diabetes, or cancer), and diagnosis of osteoporosis in themselves and their relatives were asked; in the second part, a researcher- made questionnaire regarding knowledge, attitude, and practice about the prevention and control of osteoporosis was used. The knowledge questionnaire consisted of 14 items with yes/ no / I don’t know scales (10 items for nutrition, 2 items for physical activity, and 2 items for smoking). Correct answers received score 1, incorrect and I don’t know answers received score 0. Attitude toward the effectiveness of osteoporosis prevention recommendations assessed by 11 items (6 items for nutrition, 3 items for physical activity, and 2 items for smoking) with a scale of low (score = 1), moderate (score = 2), high (score = 3), and seven items were designed for practice (5 items for nutrition, 1 item for physical activity, and 1 item for smoking). To measure the practice, we asked the participants to answer the question of how many days of the past week they did each of the seven behaviors (scores for each item were 0–7), including consumption of dairy products, fruits and vegetables, fish, and calcium and vitamin D supplements, regular walking, and avoidance of smoking.

Before the intervention and two months after it, the questionnaires were completed by the participants at their home. If the participants did not understand the meaning of some questions, the researcher explained the meaning of those questions to the participants. For illiterate participants, the questionnaires were completed by a trained health care provider through 20 minutes’ face-to-face interviews.

### Validity and reliability

Content and construct validity of the questionnaire were evaluated and confirmed through a panel of experts including seven health education specialists, a gerontologist, two health care providers, and two health volunteers from the selected villages. The internal consistency of the questionnaires was measured by Cronbach’s alpha, and values between 0.85 and 0.97 were obtained, indicating the acceptable reliability of the questionnaire. External reliability of the questionnaire was also evaluated by test-retest method over a two-week interval on a sample of 30 elderly women. Pearson correlation coefficient was obtained 0.73, indicating appropriate external reliability of the questionnaire.

### Analytic strategy

All statistical analyses were performed using the SPSS 25. The normality assumption of the variables was assessed and confirmed by the Kolmogorov-Smirnov test (P > 0.2), descriptive and Chi-square analyses were used to assess the characteristics of the samples. In order to compare the mean scores of the variables, we used independent sample t-test for between group comparisons and paired t-test for within group comparisons. The significance level was set at < 0.05.

## Results

The mean age of the participants in the control and intervention groups was 65.70 ± 7.87 and 67.05 ± 5.33 years, respectively (p = 0.161). Table 1 shows that demographic variables were not significantly different between the intervention and control groups.

**Table 1:** Distribution of participants by demographic characteristics

variable		Control group N (%)	Intervention group N (%)	p
Education levels	Illiterate	83 (80.6)	80 (81.6)	0.971
	Elementary	14 (13.6)	13 (13.3)	
	High school	6 (5.8)	5 (5.1)	
Underlying chronic diseases (hypertension, diabetes or cancer)	yes	65 (63.1)	65 (66.3)	0.371
	No	38 (36.9)	33 (33.7)	
Diagnosed with osteoporosis in the relatives	yes	82(50.8)	71(35.3)	0.572
	No	21(10.4)	27(13.4)	
Diagnosed with osteoporosis by themselves	yes	65(32.3)	65(32.3)	0.371
	No	38(18.9)	33(16.4)	

### Knowledge

The total knowledge mean score (possible range = 0–14) of all women who participated in the study was 7.85 ± 3.30. Based on inter-group comparisons, there were no significant differences in the mean scores of total knowledge, knowledge about nutrition (p = 0.447), physical activity (p = 0.126), and smoking (p = 0.157) prior to the intervention, between the control and intervention groups. However, after the intervention, the mean scores of total knowledge (p < 0.001), knowledge about nutrition (p < 0.001), physical activity (p = 0.038), and smoking (p = < 0.001) were significantly higher in the intervention group compared to the control group. Intra-group comparisons showed a significant increase in the mean score of total knowledge (p < 0.001), knowledge about nutrition (p < 0.001), physical activity (p = 0.001), and smoking (p = 0.001) in the intervention group after the intervention. However, there was no significant difference in the control group (Table 2).

**Table 2:** comparing mean scales of the study variables before and after the intervention between and groups

Variable		Possible range	Time	Group		P-value*
				Experiment Mean ± SD	Control Mean ± SD	
Knowledge	Total	0–14	Before	7.75 ± 2.58	7.95 ± 2.02	0.548
			After	10.38 ± 1.92	7.94 ± 1.95	< 0.001
	P-value**			< 0.001	0.163	
	Nutrition	0–10	Before	5.41 ± 1.96	5.61 ± 1.61	0.447
			After	7.48 ± 1.45	5.59 ± 1.55	< 0.001
	P-value**			< 0.001	0.054	
	Physical activity	0–2	Before	1.2 ± 0.68	1.36 ± 0.35	0.126
			After	1.72 ± 0.66	1.35 ± 0.67	0.038
	P-value **			< 0.001	0.7	
	Smoking	0–2	Before	1.33 ± 0.68	0.99 ± 0.73	0.157
			After	1.44 ± 0.61	0.98 ± 0.72	< 0.001
	P-value **			0.001	1	
Attitude	Total	11–33	Before	18.22 ± 2.66	17.57 ± 3.89	0.170
			After	20.52 ± 3.09	16.99 ± 3.73	< 0.001
	P-value **			< 0.001	< 0.001	
	Nutrition	6–18	Before	9.05 ± 1.90	8.87 ± 2.38	0.062
			After	10.75 ± 2.15	8.74 ± 2.37	< 0.001
	P-value **			< 0.001	0.67	
	Physical activity	3–9	Before	5.02 ± 1.15	5.22 ± 1.53	0.73
			After	8.83 ± 1.34	4.98 ± 1.17	< 0.001
	P-value **			< 0.001	0.062	
	Smoking	2–6	Before	3.54 ± 1.15	3.33 ± 1	0.053
			After	4.03 ± 1.08	3.28 ± 1.01	< 0.001
	P-value **			< 0.001	0.063	
Practice	Total	0–49	Before	11.17 ± 5.51	12.44 ± 5.22	0.094
			After	13.10 ± 5.26	12.68 ± 5.15	0.569
	P-value **			< 0.001	0.053	
	Nutrition	0–35	Before	9.40 ± 4.67	10.45 ± 4.46	0.105
			After	10.97 ± 4.57	10.67 ± 3.30	0.62
	P-value **			< 0.001	0.058	
	Physical activity	0–7	Before	1.73 ± 1.79	1.91 ± 1.44	0.44
			After	2.09 ± 1.53	1.93 ± 1.45	0.44
	P-value **			0.001	0.61	
	Smoking	0–7	Before	0.03 ± 0.17	0.07 ± 0.41	0.29
			After	0.03 ± 0.17	0.08 ± 0.41	0.29
	P-value **			1.00	0.98	

* independent sample t-test, ** paired sample t-test

### Attitude

The total attitude mean score (possible range = 11–33) of all women who participated in the study was 17.89 ± 3.75. Inter-group comparisons of the mean score of total attitude (P = 0.170) and its subscales including attitudes toward nutrition (p = 0.062), physical activity (p = 0.73), and smoking (p = 0.053) showed no significant differences between the two groups, before the intervention; however, the total mean score of attitude (P < 0.001), attitudes toward nutrition (p < 0.001), physical activity (p < 0.001), and smoking (p < 0.001) was significantly higher in the intervention group compared to the control group, after the intervention. Intra-group comparisons revealed that unlike the control group, the intervention group showed a significant increase in total mean scores of attitude (p < 0.001), attitude toward nutrition (p < 0.001), physical activity (p < 0.001), and smoking (p < 0.001) after the intervention (Table 2).

### Practice

The total attitude mean score (possible range = 0–49) of all women who participated in the study was 11.82 ± 5.38. As shown in Table 2, in inter-group analysis, there were no significant differences in the total mean score of osteoporosis prevention behaviors, practices in nutrition, physical activity, and smoking between the two study groups, both before and after the intervention (p > 0.05). In intra-group analysis, it was determined that, unlike the control group, the intervention group displayed a significant increase in the total mean scores of total practice (p < 0.001), nutritional practices (p < 0.001), and physical activity (p = 0.001) after the intervention; however, it was not enough to make a significant difference with the control group. According to the results of this study, no significant difference was observed in smoking behaviors in either intervention or control groups before and after the intervention.

## Discussion

Health communication campaign is a type of media campaigns that seeks to promote public health by developing educational health interventions. The purpose of such campaigns is to increase the individual’s awareness about the impacts of diseases and to provide them with more information regarding prevention methods [13, 18]. The present study aimed to investigate the effect of an osteoporosis prevention campaign on knowledge, attitude, and practices of elderly women towards osteoporosis in Fasa, Iran.

In the same line with the studies of Parandeh et al. (2019) in Iran [19], Oumer et al. (2020) in China [20], and Nohra et al. (2022) in Lebanon [21], in this study, the total knowledge score of the participants was assessed at a moderate level. However, Senthilraja et al. (2019) [22] and Hussein & Wahdan (2021) [23] reported that the knowledge scores of women were poor and very poor in India and Egypt, respectively.

According to the results, educational interventions increased the mean score of knowledge about the prevention and control of osteoporosis in the intervention group compared to the control group. Consistent with our study, some other studies have also shown an increase in the knowledge of participants about osteoporosis following educational intervention in Iran [19] and South Korea [24].

The results about the mean attitude score of the participants showed that elderly women had a relatively favorable attitude towards the prevention of osteoporosis. Parandeh et al. (2019), reported that the perceived benefits of Iranian middle-aged (30–45 years old) women about the role of diet and physical activity in preventing osteoporosis were higher than moderate [19]. The findings of Oumer et al. (2020) in China [20] also revealed that more than 74%, 75%, and 48% of the participants believed that physical activity, diet, and smoking cessation were effective in prevention of osteoporosis, respectively. However, Ungan & Tumer (2001) stated that elderly women displayed no proper attitude towards prevention of osteoporosis [25]. This difference may be due to the activities conducted through the past 20 years in Iran such as osteoporosis mounting public education campaings in world osteoporosis days, and and care services of several osteoporosis specialty clinics which have been developed in recent years [26].

The mean score of attitudes about osteoporosis prevention behaviors in the intervention group increased significantly more than the control group in this study. Several studies in Iran such as those of Parandeh et al. (2019) [19], Shafieinia et al. (2016) [27], and Hazavehi et al. (2007) [28] and studies in other countries including Parrott et al. [29] in the United States (2008), and Pinar and Pinar (2020) in a rural population in Turkey [30] also showed the positive effect of educational intervention on the attitudes and motivations of the participants towards osteoporosis prevention.

Consistent with the study of Nohra et al. (2022) in Lebanon [21], the findings of the present study revealed that the mean score of the participants’ practices toward preventing osteoporosis was relatively low. This is inconsistent with the findings of Ahn and Oh (2021) in South Korea [24] which reported that nutritional behaviors and physical activities in women at early old age were at moderate level and above. On the other hand, while, consistent with the studies of Ahn and Oh (2021) [24] and Parandeh et al. (2019) [19], the results of our study indicated that the mean score of overall practices as well as the participants’ practices in adherence to nutritional and physical activity advices to prevent osteoporosis increased significantly in the intervention group. In line with some other studies [29, 31, 32], this increase was not enough to make a significant difference with the control group. Furthermore, educational intervention in the present study did not significantly change tobacco use; however, given that the participants in the study were women, and a few of them (3.5%) had reported tobacco use at baseline, there was little expectation about changing their practice in this regard.

### Limitations

This is one of the few studies that was designed and implemented completely based on the principles of conducting health communication campaigns, especially for osteoporosis, but it had some limitations, one of the most important of which is that most of the participants were illiterate and the study audience was only rural elderly women, so the results may be not generalizable to the urban, male and more literate populations. Since the recommended behaviors of the participants were not observed by researchers, and their adherence to these behaviors was assessed using self-reporting questionnaire, it should be considered as another limitation of the study.

## Conclusion

In conclusion, the results of the present study suggest that health communication campaign improved knowledge, attitude, and practice of the participants towards osteoporosis. Given that the primary target group in this study was the elderly women, one of the most important challenges in this study was communicating information to participants who almost all of whom were illiterate or low-literate. The results of this study showed that in developing countries such as Iran, where most of the elderly people are illiterate, developing and implementing educational programs in the form of health communication campaigns using different educational methods, engaging secondary audience who influence the low-literate elderly, and developing educational materials appropriate to educational levels of audience through careful analysis of the audience can be an effective way to improve their knowledge, attitude and behavior. On the other hand, the designed questionnaire was a good tool to collect the necessary information in this field in the population with low education level, and the participants communicated well with it, so it can be used in similar studies.

## Data Availability

Data used in the analysis as well as all programs used for the analysis may be obtained by contacting the contacting the corresponding author on reasonable request.
